# Performance of Porous Slabs Using Recycled Ash

**DOI:** 10.3390/polym13193319

**Published:** 2021-09-28

**Authors:** Taha Awadallah El-Sayed

**Affiliations:** Structural Engineering Department, Faculty of Engineering at Shoubra, Benha University, Cairo 11629, Egypt; taha.ibrahim@feng.bu.edu.eg

**Keywords:** rice straw ash, wheat straw ash, recycled pervious concrete, mix design, permeability, porosity, pervious concrete slabs, ansys 2019-R1

## Abstract

Permeable concrete is an environmentally friendly material that improves water permeability and slip resistance. The manuscript describes a new study aimed at improving the strength of permeable concrete obtained using local materials for the partial replacement of cement with rice and wheat straw ash due to the high amount of silica and pozzolanic characteristics present in the ash. For this purpose, nine concrete mixes were made (Phase I). The mixes were classified into four groups: Group A, with cement/aggregate ratios of 0.23, 0.34, and 0.44 for Mixes 1, 2, and 3, respectively; Group B, with sand added at 10% and 15% to the coarse aggregate for Mixes 4 and 5; Group C, with rice straw ash replacement ratios of 10% and 15% in the cement for Mixes 6 and 7; and, finally, Group D with wheat straw ash replacement ratios of 10% and 15% in the cement for Mixes 8 and 9. For Groups B to D, the water/binder ratio was 0.238. Fresh and hardened concrete tests were conducted. The results showed that Mixes C and D, which contained rice and wheat straw ash, increased the compaction factor due to their spherical shape and higher surface area compared with traditional pervious concrete. Additionally, permeability and porosity increased slightly for the mixes using rice and wheat straw ash. This could be attributed to increasing the interconnected voids. Optimum porosity was reached with 15% rice straw ash. The optimum mix design from Phase I was used in Phase II. Therefore, six pervious concrete slabs, reinforced with different types of reinforcement, were tested under flexural load. With the help of ANSYS, a finite element model was created to verify the results of experiments. The results of the numerical simulation are consistent with the results of the experiment. This article represents a definite step to new knowledge in the field of research of permeable concrete obtained using the partial replacement of cement with rice and wheat straw ash. Hence, this form of concrete can be used for parking lot paving, sludge beds for sewage plants, swimming pool surfaces, bridge walkways, zoo area floors, and animal barns. This concrete can also be used in applications requiring lightweight concrete.

## 1. Introduction

Pervious concrete is a concrete category that was developed for reducing the runoff amount of water and improving water flow near sidewalks and parking lots. This type of concrete can be produced by mixing coarse aggregate, cement, and water with/or without admixtures [1]. Pervious concrete is relatively inexpensive due to the lower cement content in the mix. The lack of fine aggregates decreases the surface to be covered with cement paste. Due to the pervious concrete components, there is a thin layer of cement paste and a higher void content in comparison to traditional concrete. 

These voids occupy about 15–30% of the total volume [2] and are vital in specifying the characteristics of that concrete, particularly its compressive strength and permeability [3]. As the void content increases, lower compressive strength and higher permeability results [4,5]. Due to its lower compressive strength, this concrete is used as a building material with limited usage [6]. Pervious concrete has a high permeability that is desired, with negligible compressive strength. To improve compressive strength, many investigators have substituted the coarse aggregate with precise fine materials [7,8,9]. These studies showed a significant increase in concrete strength with reduced observed concrete permeability.

Many researchers have enhanced the pervious concrete cement paste strength by increasing the ratio of cement/aggregate, reducing the aggregate size, or reducing the ratio of water/cement [10,11,12,13,14,15]. Results showed that permeability and porosity were decreased by increasing the used binder and size of aggregate. Pervious concrete has almost no slump and a lower compaction factor. To enhance workability and strength, chemical admixtures should be used, such as superplasticizers and polymers [16,17,18]. Butta and Merza [13] studied polymers for improving pervious concrete properties. The results showed enhancement in compressive, flexural strength, and workability when using admixtures. 

Poor permeability coefficients and void ratio results were also obtained. Similar results were stated by many researchers when using superplasticizers [8,9]. Giustozzi [19] examined the behavior of pervious concrete with three different polymers. The results showed that the addition of polymers increased strength and durability and delayed the curing time. The best behavior was obtained when using polyvinyl acetate polymer. 

Huang et al. [20] studied superplasticizers to improve pervious concrete properties. They observed decreases in permeability when using latex only, and further decreases were obtained when adding fine aggregate. On the other hand, compressive and tensile strength were increased when using superplasticizers, with/without fine aggregate, and fiber with a 0.35 w/c ratio for all mixes. The same trend was obtained by others [21,22] with variable superplasticizer ratios and a constant w/c. Chen et al. [23] revealed the four main items that impacted the pervious concrete strength: porosity, the water/cement ratio, characteristics of cement paste, and coarse aggregate size and volume. 

Therefore, silica fume and superplasticizers can be added to the mix to improve the strength and workability of pervious concrete [24]. Other cement replacement materials can be added to the concrete mix, such as nanomaterials, silica fume, rice husk ash, rice straw ash, and wheat straw ash, for a better pozzolanic reaction [25,26,27,28,29,30,31,32,33,34,35,36,37]. They concluded that using recycled materials affects the permeability and compressive strength as follows. The result also showed that using these cement replacement materials improved the compressive and tensile strength of concrete specimens. Rice husk, rice straw, and wheat straw ashes gave a 10–20% increase in compressive and tensile strength with 15% cement replacement. Additionally, they provided a decrease in permeability of 10–12%. Using filler and pozzolan in concrete mixes have been extensively studied. Generally, adding filler materials provides a filler and chemical impact [38,39,40,41,42,43]. Filler primarily makes a cement paste effect, while pozzolan generates extra CSH and CAH, which make a homogeneous paste that is very dense [44,45]. The fillers improve the concrete paste, which derives from an improvement in pore structure. The number of small pores increases while, at the same time, the number of large pores decreases, which has a positive influence on strength and durability. Other pozzolanic materials may also be used, such as fly ash and diatomite [46]. Using fly ash and diatomite gives long-term increased compressive strength and increased strain paste capacity.

The aim of this study is to enhance the properties of pervious concrete produced using local cement replacement materials, such as rice and wheat straw ash. Rice and wheat straw can be transformed from agricultural waste into a cement replacement material due to the high amount of silica and pozzolanic characteristics present in the ash. 

## 2. Materials and Methods 

**Figure 1:**
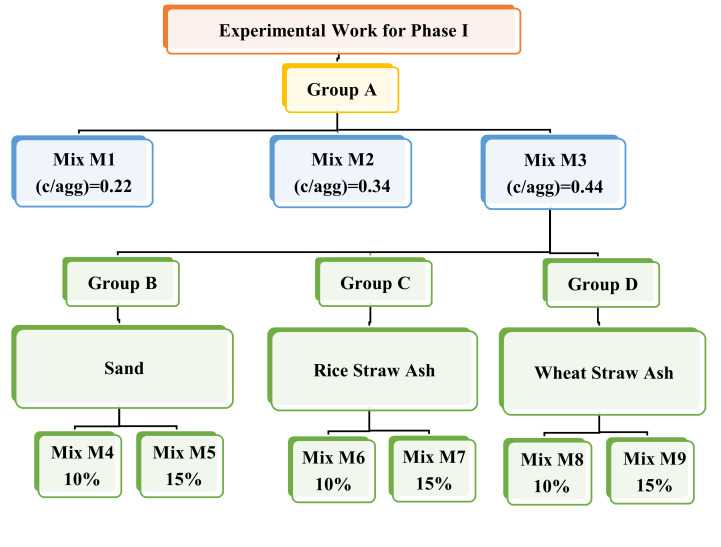
Experimental program for Phase I.

Figure 1 and Table 1 illustrate the 9 mix proportions with 162 samples used in this study. The mixes were classified into four groups, Groups A–D. Group A, with cement/aggregate ratios of 0.23, 0.34, and 0.44 for Mixes 1, 2, and 3, respectively; Group B, with sand added at 10% and 15% to the coarse aggregate for Mixes 4 and 5; Group C, with rice straw ash replacement ratios of 10% and 15% in the cement for Mixes 6 and 7; and, finally, Group D with wheat straw ash replacement ratios of 10% and 15% in the cement for Mixes 8 and 9. For Groups B to D, the water/binder ratio was 0.238. The variables were the ratio of cement paste to aggregate, with sand, rice, and wheat straw ash as a partial alternative ratio. All considered ratios were expressed on a dry basis. Fresh concrete tests were conducted to assess the slump, compaction factor index, and density. Hardened concrete tests were performed to obtain compressive strength, splitting tensile strength, flexural strength, permeability, and porosity. The results showed that the porosity increased for mixes using rice and wheat straw ash. 

### 2.1. Materials

#### 2.1.1. Fine Aggregate

**Figure 2:**
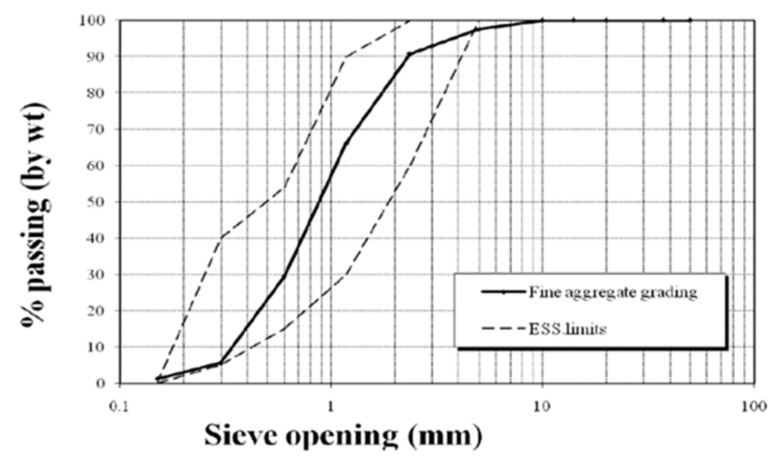
Grading curve for the fine aggregate.

Sand with a specific gravity of 2.50 kg/m^3^ and fineness modulus of 2.4, obtained from a local supplier, was used in this research. According to the Egypt Standard Specification (ESS) 203/2018 [47] and ASTM C127-88 [48], a sieve analysis was performed. The results of the sieve analysis and the physical property test results are shown in Table 2 and Figure 2.

#### 2.1.2. Coarse Aggregate 

**Figure 3:**
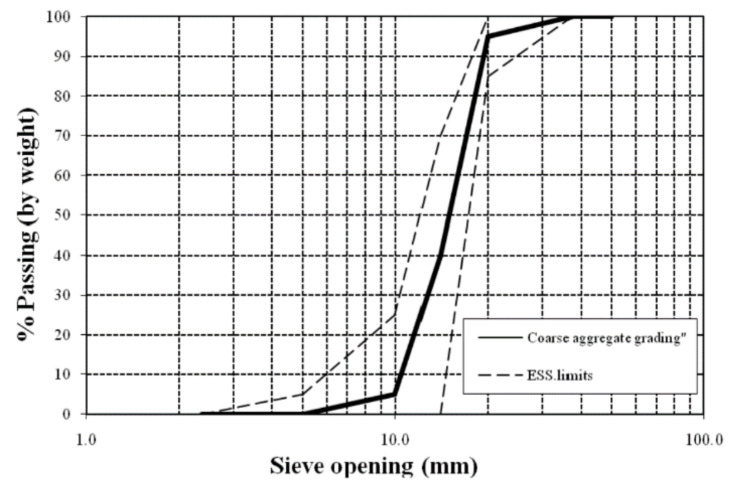
Grading curve for the coarse aggregate.

A conventional aggregate of crushed limestone with a maximum size of 10 mm was obtained from a local quarry. According to Egypt Standard Specification (ESS) 203/2018 [47] and ASTM C136-84a [49], sieve analysis was performed. The aggregate’s mechanical and physical properties are shown in Table 3, and the grading is as shown in Figure 3.

#### 2.1.3. Cement

OPC type CEM I 52.5N with a specific gravity of 3.15 kg/m^3^ complies with ASTM C150 [50]. The physical and chemical properties are given in Table 4 and Table 5, respectively.

#### 2.1.4. Recycled Rice Straw Ash (RSA) and Recycled Wheat Straw Ash (WSA)

Rice and wheat straw ash, as forms of agricultural waste, were used as a cement replacement material with unit weights of 840 and 820 kg/m^3^, respectively. RSA and WSA were first ground in a bug milling machine [29,30] and were then passed through different sieves of 20–200 mesh sizes. The chemical compositions for RSA and WSA are given in Table 6, which fulfilled ASTM C618 [51].

#### 2.1.5. Super Plasticizer 

‘MasterGlenium 51′ superplasticizer was provided by BASF. The superplasticizer density was 1.1 kg/L. The chloride content was less than 0.1%, and the alkaline content was less than 3%, which confirms Bong et al. [52].

#### 2.1.6. Polypropylene Fibers

Used in amounts of 0.1% and 0.2% by cement weight. The physical and mechanical properties are given in Table 7.

#### 2.1.7. Geogrid

Geogrid CE121 polyethylene of two types were used: Type I, with opening dimensions of 50 × 50 mm, and type II, with opening dimensions of 70 × 70 mm.

#### 2.1.8. GFRP Bars 

Glass fiber reinforced polymer bars with mechanical properties, as given in Table 8.

### 2.2. Description of Specimens for Phase I

Table 1 and Figure 1 present the nine mixes divided into four groups, A–D. The specimens were kept in the molds for 24 h in air and then removed from the molds and immersed in fresh water until the test time.

### 2.3. Description of Specimens for Phase II

**Figure 4:**
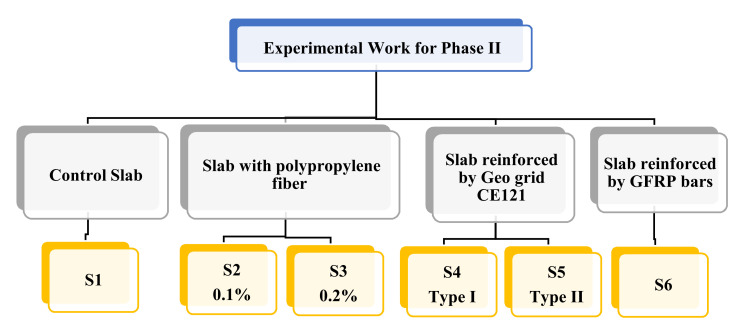
Experimental program for Phase II.

Table 9 presents the slabs mix design and Figure 4 shows the used six group slabs, S1–S6. The slabs were kept in the molds for 24 h in the air, and then removed from molds and cured in freshwater until the test time.

### 2.4. Experimental Tests for Phase I

The tests executed were the slump, compacting factor, density, compressive strength, split tensile strength, flexural strength, permeability, and porosity tests.

#### 2.4.1. Slump Test

A concrete slump test was executed according to ASTM C143 [53]. The slump was measured as the difference between the mold height and the concrete height after removing the mold. For all mixes, nine samples (nine samples/mix) were tested.

#### 2.4.2. Compacting Factor Test

The compacting factor test was done according to ACI Standard 211.3 [54]. Equation (1) was used for calculating the compacting factor value. For all mixes, nine samples (nine samples/mix) were tested.

(1) $$F=\frac{\;weight\;of\;partially\;compacted\;concrete}{weight\;of\;fully\;compacted\;concrete}$$

#### 2.4.3. Density

Cylindrical specimens with dimensions of 150 × 300 mm were used in measuring the density according to ASTM C138 [55] using Equation (2). For all mixes, nine samples (nine samples/mix) were tested.

(2) $$D=\frac{M}{V}\;$$

where *D* is the density, *M* is the sample mass, and *V* is the sample volume.

#### 2.4.4. Compressive Strength

Cubes with dimensions of 150 mm × 150 mm × 150 mm were used in measuring the compressive strength according to ASTM C39 [56] using Equation (3). We used an ELE automatic compression testing machine at 2000 kN. For each mix, three cubes were tested for 7 and 28 days.

(3) $$\mathrm{Rc}=\frac{P}{A}$$

where Rc is the compressive strength (MPa), *P* is the load (kN), and *A* is the area (mm^2^).

#### 2.4.5. Split Tensile Strength

Cylindrical specimens with dimensions of 150 × 300 mm were used in measuring the splitting tensile strength according to ASTM C496 [57]. A universal automatic testing machine of 1000 kN was used. For each mix, three cylinders were tested for 7 and 28 days. The maximum tensile stress was calculated using Equation (4).

(4) $$T=\frac{2P}{\pi dl}$$

where *P* is the vertical load applied, *l* is the cylinder length, *d* is the cylinder diameter, and *T* is the tensile stress.

#### 2.4.6. Flexural Strength

Prism samples of 100 × 100 × 500 mm were used in measuring the flexural strength according to ASTM C78 [58]. A universal automatic testing machine of 1000 kN was used. For each mix, three prisms were tested. The flexural strength is expressed as the rupture modulus *R* (MPa) using Equation (5).

(5) $$R=\frac{PL}{bd^{2}}$$

where *R* is the rupture modulus, *P* is the maximum utilized load indicated by the testing machine, *L* is the span length, *b* is the specimen width, and *d* is the specimen depth.

#### 2.4.7. Permeability

Beams of 300 × 300 × 100 m were used in determining the permeability according to ASTM PS 129 [59]. For each mix, three beams were tested. The coefficient of water permeability can be computed using Equation (6).

(6) $$K=L\frac{Q}{A(L+H)}$$

where *Q* is the water amount (cc/s); *A* is the concrete specimen cross-section area (mm^2^); *H* = (h1 − h2), h1 is the initial water level (mm) and h2 is the final water level (mm); *L* is the concrete specimen thickness (mm), and *K* is the water permeability coefficient (mm/s).

#### 2.4.8. Porosity

Cubes of 150 × 150 × 150 mm were used in determining the porosity according to ASTM D7063/D7063M [60]. For each mix, three cubes were tested. The porosity can be calculated using Equation (7).

(7) $$P=\left(1-\frac{W_{2}-W_{1}}{\rho_{w}V}\right)\;100\%$$

where *P* is total porosity (%), *W*_1_ is the oven dry weight (kg), *W*_2_ is the weight under water (kg), *V* is the sample volume (m^3^), and $\rho_{w}$ is the water density at 21 °С (kg/m^3^).

### 2.5. Experimental Tests for Phase II

Six pervious concrete slabs with 1200 × 1200 × 140 mm dimensions were tested for flexural strength using a 5000 kN universal compression testing machine connected to a data acquisition system. A 70 mm thick stiff rubber layer was placed under the slab to be like the subgrade reaction. A vertical concentrated load was applied at the center of the slab by a 125 mm radius circular plate. The slabs were instrumented to record the measured actual deformations. The deformations were monitored using LVDT connected to the data acquisition system. Table 10 summarizes all the slab details. Table 2 shows the best economical mix design for the pervious concrete slabs obtained in Phase I.

## 3. Results and Discussion

### 3.1. Slump Test Results

**Figure 5:**
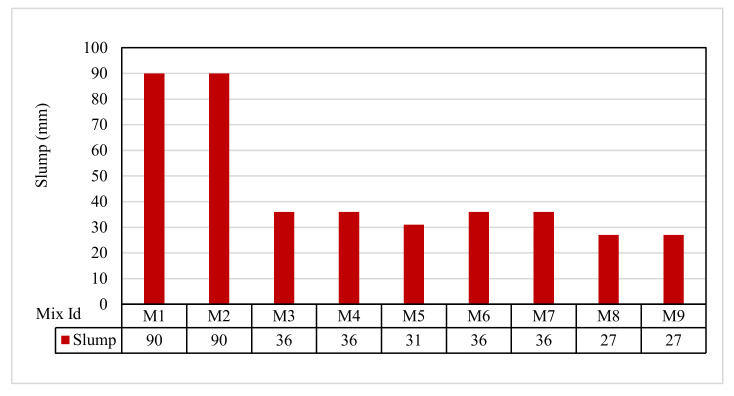
The slump test results.

The slump test results for each mix are plotted in Figure 5. The slump results ranged from 27 to 90 mm. The slump was 90 mm for mixes M1 and M2 and improved to 27–36 mm for all the other mixes, M3 to M9. This led to a stiff mix of a near-to-zero slump, which agreed with Mazumdar et al. [61].

### 3.2. Compaction Factor Test Results

**Figure 6:**
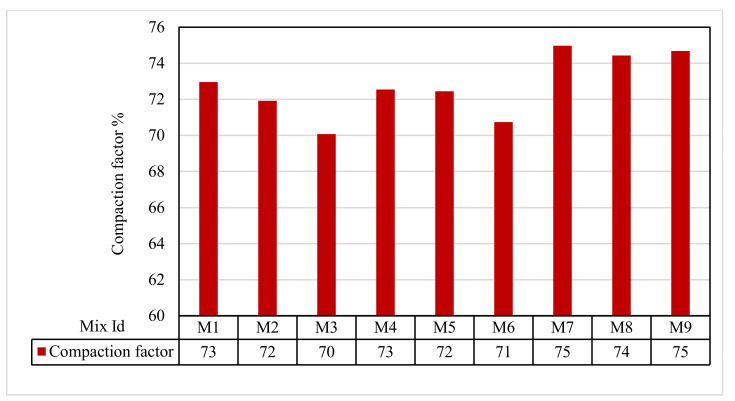
Compaction factor test results.

Figure 6 shows that the compaction factor results for all mixes ranged from 70.07% to 47.97%. The results of Group A were 72.95%, 71.91%, and 70.07%, respectively, for M1, M2, and M3. M3 in Group A was used as a control mix for all other mixes. In Group B, the compaction factors were 72.54% and 72.45%, respectively, for M4 and M5. Similarly, in Group C, for the mixes M6 and M7, the compaction factors were 70.74% and 74.97%, respectively. Finally, in Group D, for the mixes M8 and M9, the compaction factors increased by 74.43% and 74.67%, respectively.

### 3.3. Density Test Results

**Figure 7:**
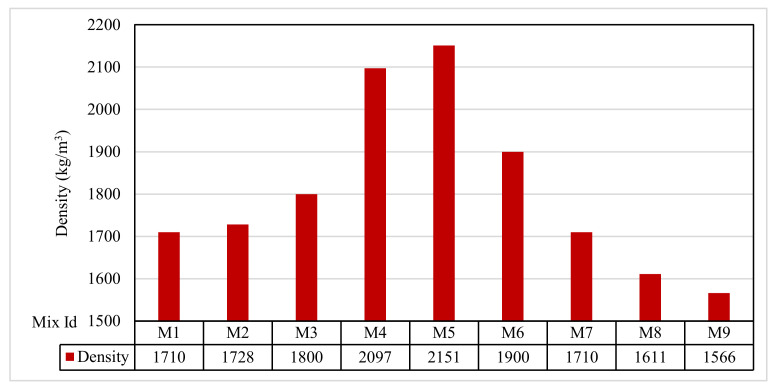
The density test results.

The density results are recorded in Figure 7 for all the mixes. M1 had a density of 1710 kg/m^3^, while mix M2 had a density of 1728 kg/m^3^. M3 had a density of 1800 kg/m^3^. For Group B, the mixes M4 and M5 had densities of 2097 and 2151 kg/m^3^, respectively. In Group C, the mixes M6 and M7 had densities of 1900 and 1710 kg/m^3^, respectively. In Group D, the mixes M8 and M9 had densities of 1611 and 1566 kg/m^3^, respectively. The results showed that the Group B mixes, M4 and M5, with fine aggregates, had a higher density due to the reduction in voids compared with the other mixes, which agreed with Mazumdar et al. [61].

### 3.4. Compressive Test Results

**Figure 8:**
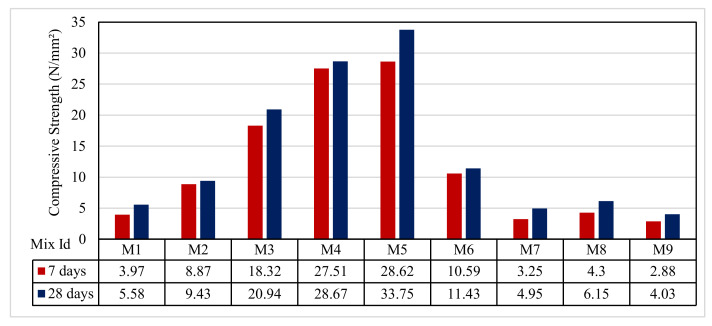
The compressive strength test results.

Figure 8 shows the compressive strength test results for the samples at 7 and 28 days of casting. The results ranged from 2.88 to 28.62 N/mm^2^, which is appropriate for a varied range of usage. For Group A, the compressive strength results at 7 days were 3.97, 8.87, and 18.32 N/mm^2^, respectively, for M1, M2, and M3, and, at 28 days, they were 5.58, 9.43, and 20.94 N/mm^2^, respectively. For Group B, the mixes M4 and M5, the compressive strength results at 7 days were 27.51 and 28.62 N/mm^2^; at 28 days, they were 28.67 and 33.75 N/mm^2^, respectively. For Group C, the mixes M6 and M7, the compressive strength results at 7 days were 10.59 and 3.25 N/mm^2^; at 28 days, they were 11.43 and 4.95 N/mm^2^. Finally, for Group D, the mixes M8 and M9, the compressive strength results at 7 days were 4.30 and 2.88 N/mm^2^; at 28 days, they were 6.15 and 4.03 N/mm^2^, respectively. 

The results showed that mixes M4 and M5 in Group B had higher strengths at 7 and 28 days. This could be attributed to the presence of fine aggregate, as shown by Mazumdar et al. [61]. The low difference in compressive strength at 7 and 28 days was due to shear failure in the aggregate, as shown by Mazumdar et al. [61]. The decrease in compressive strength for all other mixes was due to the presence of the air content, as shown in Desmaliana Erma et al. [62]. The compressive strengths measured for the mixes M6–M9 were 42% to 84% and 45% to 81% lower than those of mix M3; this might be because the quantity of RSA and WSA in the mix may be higher than that required to react with the liberated calcium hydroxide resulting from cement hydration, thus leading to excess silica being leached out and causing a deficiency in strength as it replaces part of the cementitious material but does not contribute to strength; this is in agreement with data published by Jankovský et al. [63].

### 3.5. Splitting Tensile Test Results

**Figure 9:**
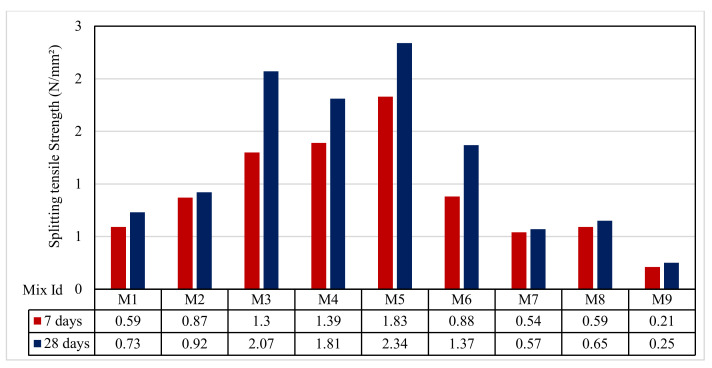
The splitting tensile strength test results.

Figure 9 shows that the splitting tensile strength ranged from 0.21 to 1.83 N/mm^2^ and from 0.25 to 2.34 N/mm^2^ for the results from 7 and 28 days, respectively. As predicted, the splitting tensile strengths at 28 days were higher than those of 7 days. For Group A, the splitting tensile strengths for the mixes M1, M2, and M3 at 7 days were 0.59, 0.87, and 1.3 N/mm^2^; at 28 days, they were 0.73, 0.92, and 2.07 N/mm^2^, respectively. For Group B, the mixes M4 and M5 were, at 7 days, 1.39 and 1.83 N/mm^2^ and, at 28 days, 1.81 and 2.34 N/mm^2^, respectively.

For Group C, the mixes M6 and M7, the splitting tensile strengths decreased at 7 and 28 days, where they were 0.88 and 0.54 N/mm^2^ as well as 1.37 and 0.57 N/mm^2^, respectively. Finally, for Group D, the mixes M8 and M9, the splitting tensile strengths at 7 days were 0.59 and 0.21 N/mm^2^ as well as 0.65 and 0.25 N/mm^2^ at 28 days, respectively. The results showed that a similar trend was observed for compressive strength. The splitting strength for the Group B mixes M4 and M5 had higher strengths at 7 and 28 days, as shown by Ali Shagea and Kacha Smit [64]. On the other hand, the low strength for the rest groups was due to the poor bond between the cement paste and aggregate, which agreed with Ali Shagea and Kacha Smit [64]. As explained earlier in the compressive strength, these splitting strengths measured for mixes M6–M9 were 32% to 84% (at 7 days) and 33% to 88% (at 28 days) lower than those of mix M3, agreeing with Jankovský et al. [63].

### 3.6. Flexural Test Results

**Figure 10:**
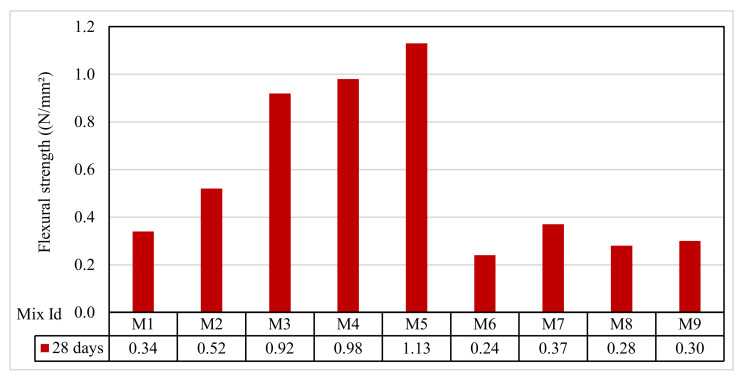
The flexural strength test results.

Figure 10 shows that the 2-day flexural strength of the tested mixes ranged between 0.24 and 1.13 N/mm^2^. Group A flexural strengths were 0.34, 0.52, and 0.92 N/mm^2^ for the mixes M1, M2, and M3, respectively. The group B flexural strengths were 0.98 and 1.13 N/mm^2^ for the mixes M4 and M5, respectively. In Group C, for the mixes M6 and M7, the flexural strengths were 0.24 and 0.37 N/mm^2^, respectively. In Group D, the mixes M8 and M9, the flexural strengths were 0.28 and 0.30 N/mm^2^, respectively. The results indicated that the mixes M4 and M5 for Group B had higher strengths at 7 and 28 days. This could be attributed to the presence of fine aggregate, as seen in Mazumdar et al. [61].

### 3.7. Permeability Test Results

**Figure 11:**
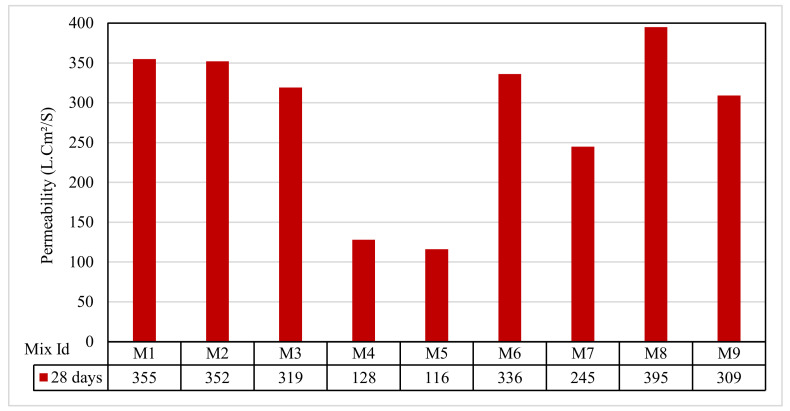
The permeability test results.

Figure 11 shows that the permeability for the concrete mixes ranged between 116 and 395 lt.cm^2^/s. The Group A permeabilities were 355, 352, and 319 lt.cm^2^/s for the mixes M1, M2, and M3, respectively. For the Group B mixes M4 and M5, the permeabilities were 128 and 116 lt.cm^2^/s, respectively. Permeability was decreased when compared with Group A. Thus, it is recommended to use sand with a percentage of less than 10% for an accepted permeability. The permeability decreased with an increase in rice straw ash by 10% and 15% in Group C for the mixes M6 and M7 from 336 to 245 lt.cm^2^/s. 

Similarly, in the Group D mixes M8 and M9, the permeability decreased with an increase in wheat straw ash replacement by 10% and decreased with an increase of 15% from 395 to 309 lt.cm^2^/s. Using the rice and wheat straw ash in Groups C and D, respectively, increased the permeability compared with Group A more than with Group B, which agreed with Narayana et al. [65]. The permeability of the pervious samples increased directly with increases in the interconnecting voids, which agreed with Kia et al. [66]. 

### 3.8. Porosity Test Results

**Figure 12:**
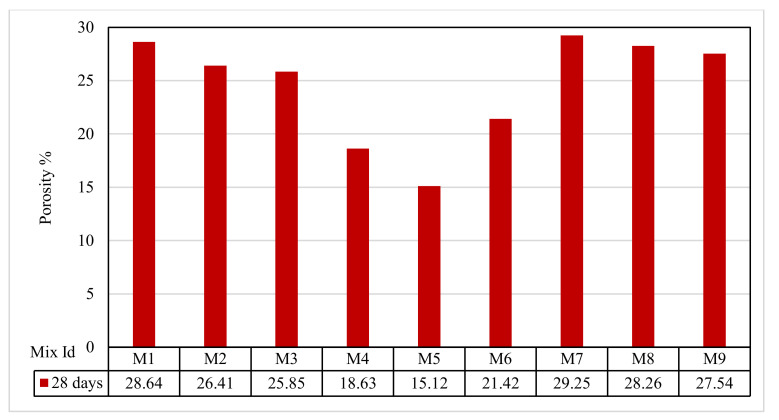
The porosity test results.

The porosity of the tested mixes ranged from 15.12% to 29.25%, as shown in Figure 12. Group A showed almost the same porosity, with values of 28.64%, 26.41%, and 25.85% for the mixes M1, M2, and M3, respectively. For the Group B mixes M4 and M5, the porosities were 18.63% and 15.12%, respectively. The Group C porosities were 21.42% and 29.25%, respectively. For Group D, the porosities were 28.26% and 27.54%. The porosity increase was because of the increase in the interconnected voids when the percentage of the RSA and WSA replacement increased, which agreed with Narayana et al. [65]. When the interconnected voids increased, extra water content was able to pass through the concrete, and, therefore, the concrete permeability directly increased, which agreed with Kia et al. [66].

### 3.9. Ultimate Load and Displacements

**Figure 13:**
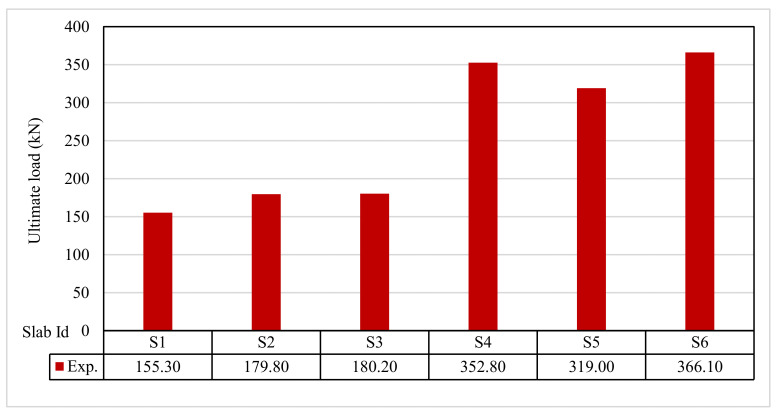
Ultimate load for tested slabs.

**Figure 14:**
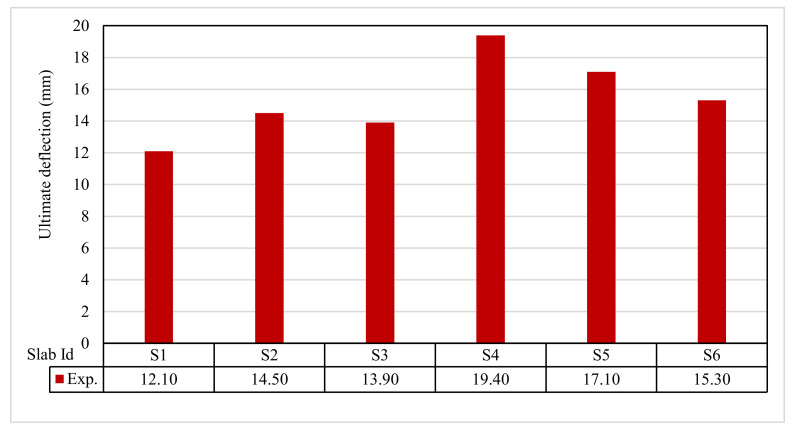
Ultimate defection for tested slabs.

The ultimate load and displacements for all the slabs are given in Table 11. Figure 13 shows that the ultimate load ranges between 155.30 and 366.10 kN, and the ultimate displacement ranges between 12.10 to 19.40 mm, as indicated in Figure 14.

Results show an increase in the ultimate load and displacement for all reinforcement types than control slabs. Using 0.1% and 0.2% polypropylene fibers provided an insignificant increase in the ultimate load by 179.80 and 180.20 kN, respectively. Using geogrid type I and II gave the ultimate loads of 352.80 and 319.00 kN, respectively. GFRP bar slabs exhibited the highest ultimate failure load of 366.10 kN. The impact of RSA with 15% on slab load-carrying capacity is clear from the result. This could be attributed to the presence of RSA, as seen in the study by Mazumdar et al. [61].

### 3.10. Ductility Ratio and Energy Absorption

**Figure 15:**
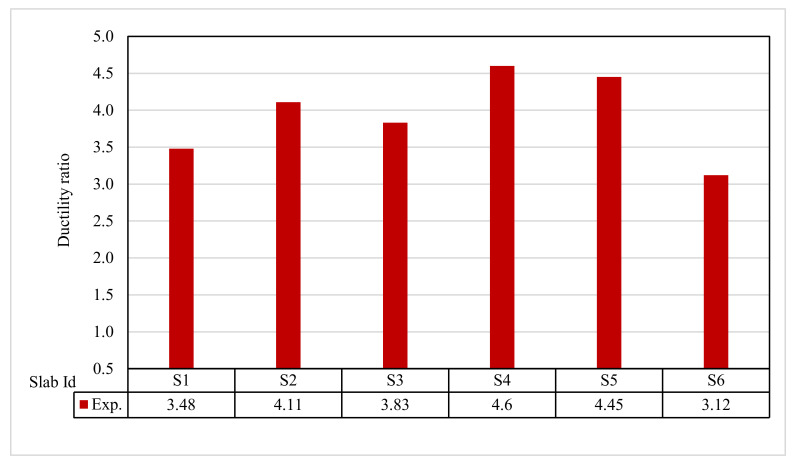
Ductility ratio for tested slabs.

**Figure 16:**
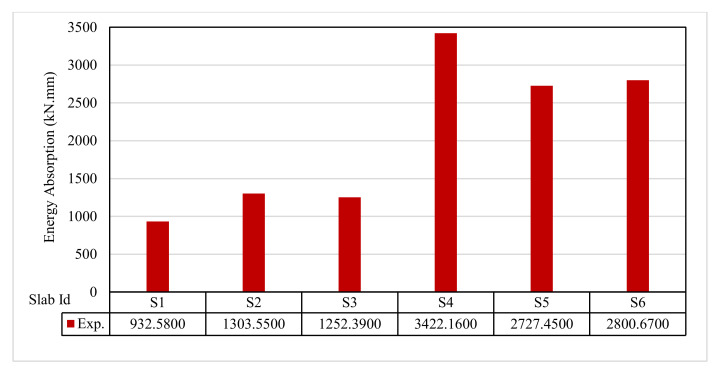
Energy absorption for tested slabs.

Figure 15 and Figure 16 show the ductility ratio and energy absorption for the tested slabs. The ductility ratio ranged from 3.12 to 4.60. The ductility ratio was reduced by using GFRP bars. In contrast, most of the slabs incorporating other reinforcements achieved significant deflection at failure. The energy absorption of S6 was lower than the control S1. The energy absorption was about 139.78% for S2, 134.29% for S3, 366.97% for S4, 292.46% for S5, and 300.31% for S6 relative to control slab S1.

### 3.11. Load-Deflection Relationship

**Figure 17:**
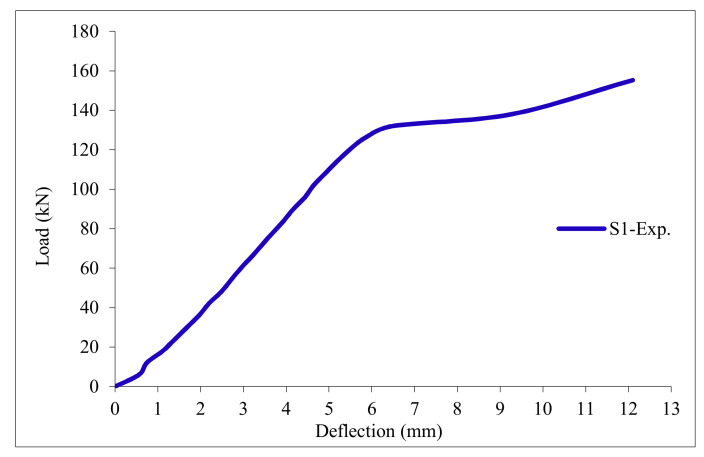
Load deflection curve for control slab S1.

**Figure 18:**
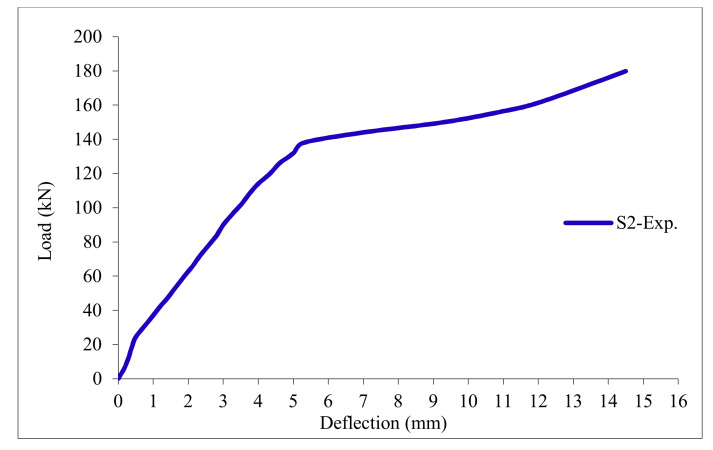
Load deflection curve for slab S2.

**Figure 19:**
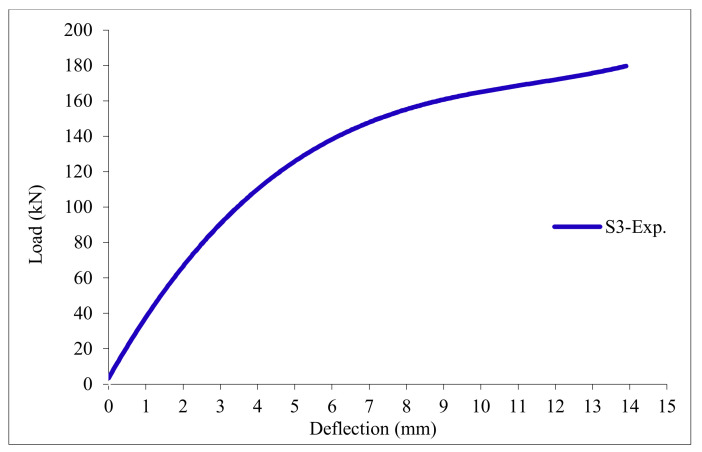
Load deflection curve for slab S3.

**Figure 20:**
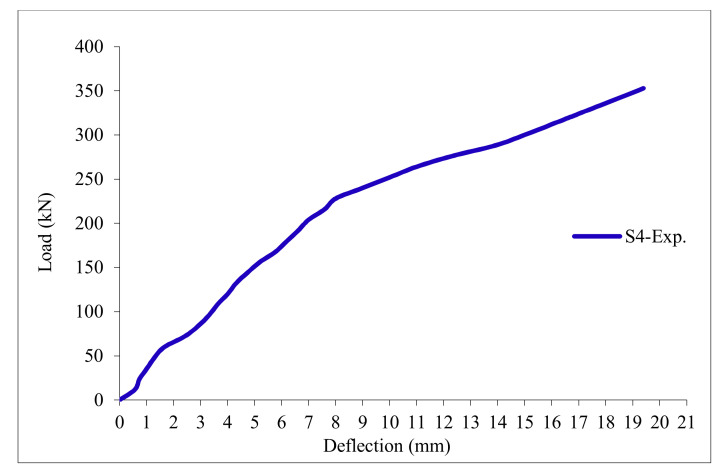
Load deflection curve for slab S4.

**Figure 21:**
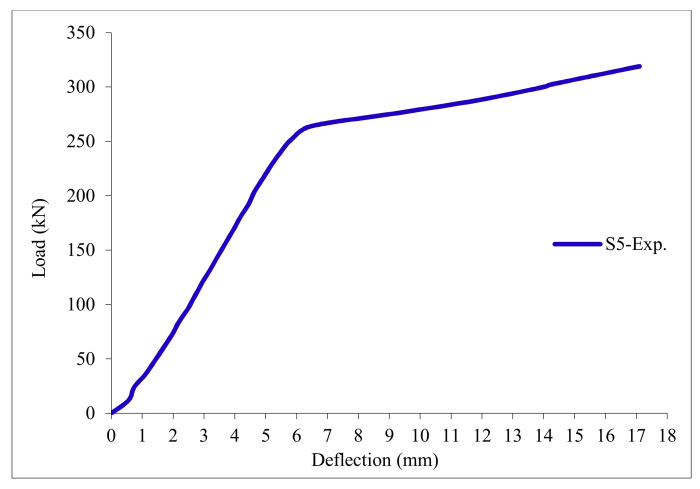
Load deflection curve for slab S5.

**Figure 22:**
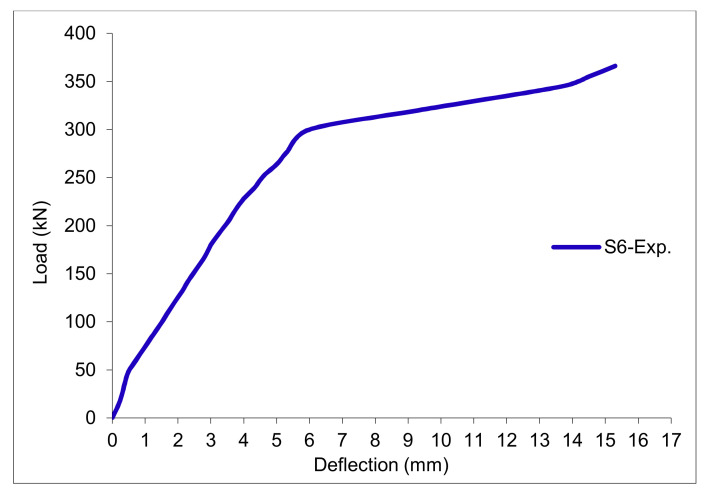
Load deflection curve for slab S6.

The load-deflection curves are plotted in Figure 17, Figure 18, Figure 19, Figure 20, Figure 21 and Figure 22 for all slabs. Figure 17 shows the load-deflection curve for the control slab. From these figures, it can be clearly seen that for all slabs, the relationship between the load and deflection can be divided into two stages: Stage 1 is the elastic behavior until first cracking—The load-deflection relationship in this stage is linear; Stage 2, with large plastic deformation. Figure 18 and Figure 19 show the load-deflection curves for slabs S2 and S3, reinforced by polypropylene 0.1% and 0.2%. Figure 20 and Figure 21 show the load-deflection curves for slabs S4 and S5 that are reinforced by geogrid Type I and Type II. Figure 22 shows the load-deflection curve for slab S6, reinforced by GFRP bars.

The results show an increase in ultimate failure load and ultimate displacement for all slabs compared to control slab S1. Slabs S2 and S3 increased the ultimate load by 115.78% and 116.03% compared to S1. Therefore, it can be concluded that the increase in fiber reinforcing causes a noticeable increase in the ultimate load and ultimate displacement, which agrees with Erfan et al. & Roesler et al. [67,68]. In slabs S4 and S5, the ultimate load increased by 227.17% and 205.41%, respectively. Similarly, for slab S6, reinforced by glass fiber rods, the ultimate load increased by 235.74% compared with slab S1, which agreed with Mahroug et al. [69]. The ultimate displacements ranged from 12.10 to 19.40 mm for all tested slabs. Slab S1 had the lowest displacement value, and slab S4 gave the highest displacement. Hence, it can be said that slabs with geogrid type I had more ductile behavior. The impact of RSA with 15% on slab load-carrying capacity is clear from the result.

### 3.12. Crack Patterns

The cracks developed for all slabs under flexural load as vertical cracks near the slab’s edges, starting from the bottom. The cracks are enlarged near the compression zone. The slabs are collapsed by diagonal cracks under the loading plate. All slabs exhibited almost the same crack.

## 4. NonLinear Finite Element Analysis

A finite element model was created to validate the experimental program using the ANSYS 2019-R1 [70] NLFEA program.

### Modeling

**Figure 23:**
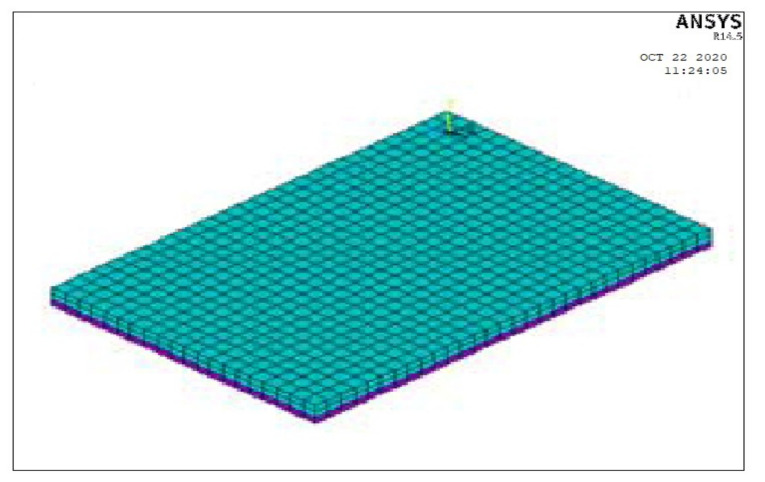
Finite element model.

Finite element analysis was carried out to investigate the flexural behavior of pervious concrete slabs reinforced with different types of reinforcement, as in Table 12. The ANSYS-2019-R1 finite element model is indicated in Figure 23. The investigated behavior includes the crack pattern, the ultimate load, and deflection of the slabs.

## 5. Comparisons between Experimental and NLFEA Results

There was good agreement obtained between experimental and ANSYS 2019-R1 results by applying the finite element model. Comparisons were applied between ultimate load, deflection at ultimate load, the first crack load, and the crack patterns.

### 5.1. Ultimate Load

**Figure 24:**
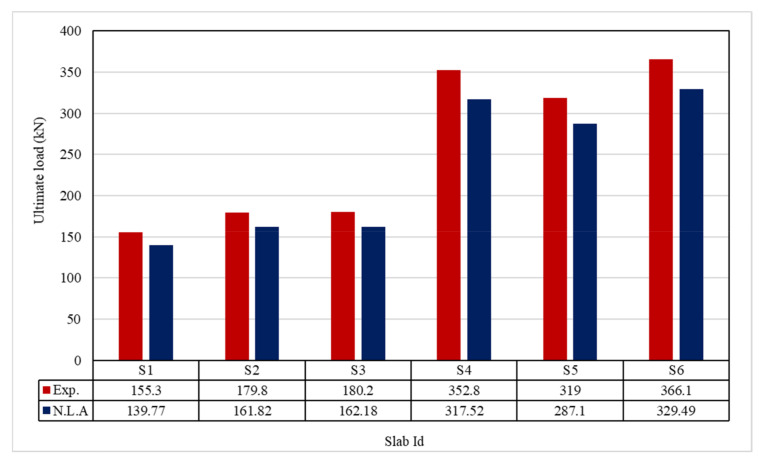
Comparison between Exp. and NLA ultimate loads.

Table 13 and Figure 24 shows the comparison between the ultimate experimental load and the numerical one. There is a reasonable agreement between the experimental and numerical ultimate loads.

### 5.2. Ultimate Displacement

**Figure 25:**
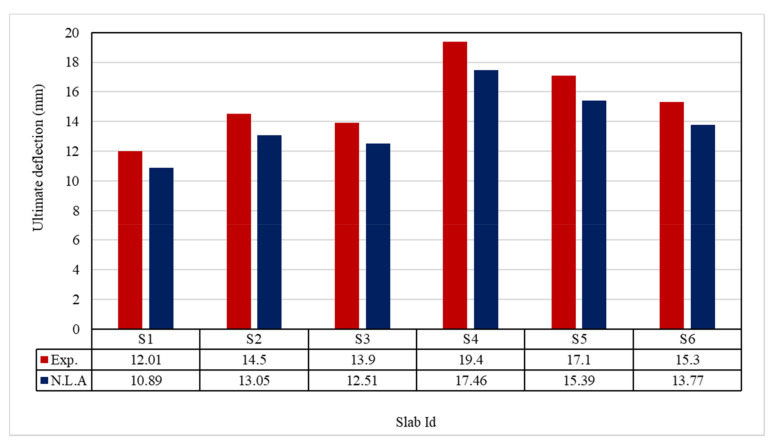
Comparison between Exp. and NLA ultimate deflections.

**Figure 26:**
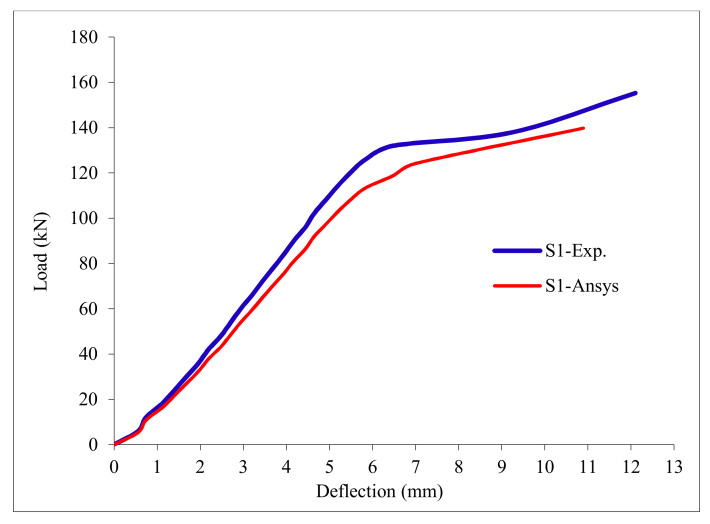
Load deflection curve for control slab S1.

**Figure 27:**
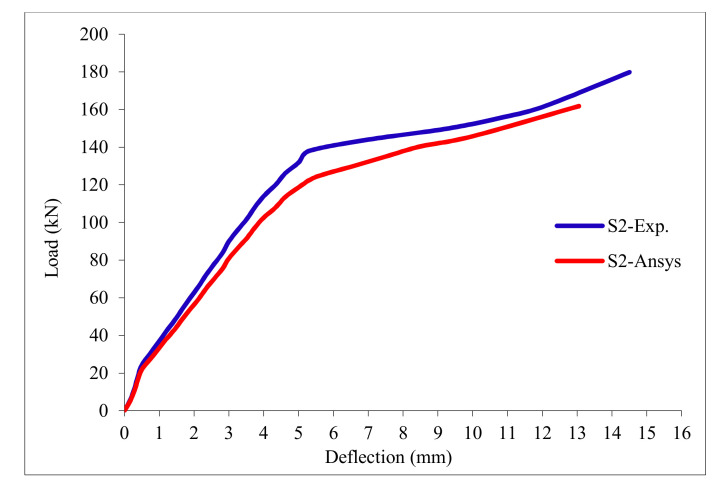
Load deflection curve for control slab S2.

**Figure 28:**
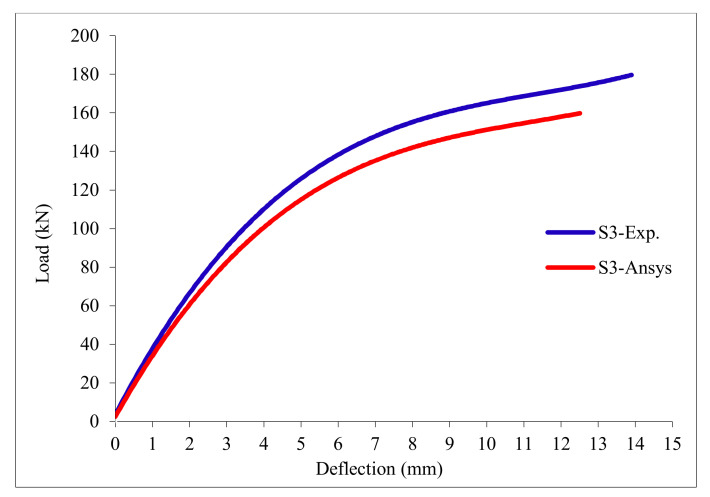
Load deflection curve for control slab S3.

**Figure 29:**
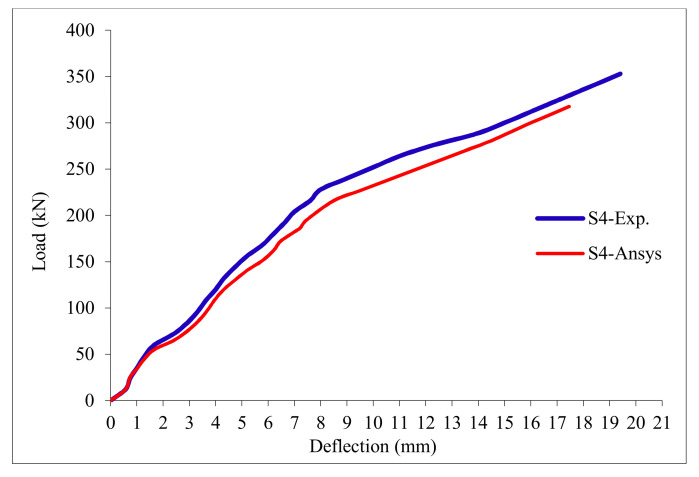
Load deflection curve for control slab S4.

**Figure 30:**
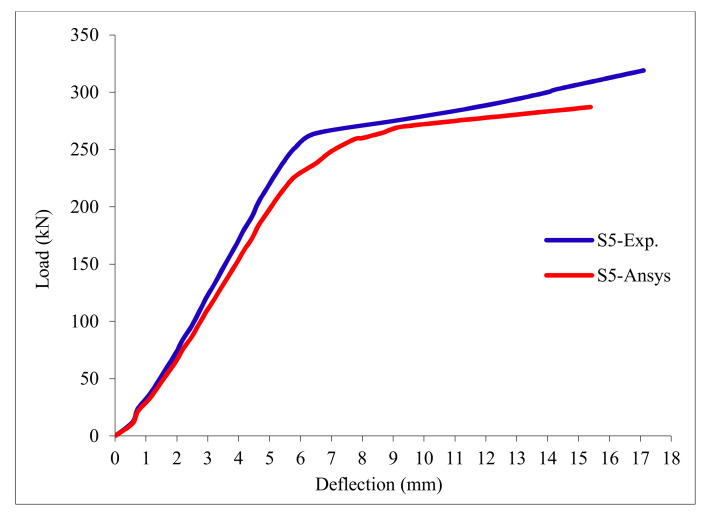
Load deflection curve for control slab S5.

**Figure 31:**
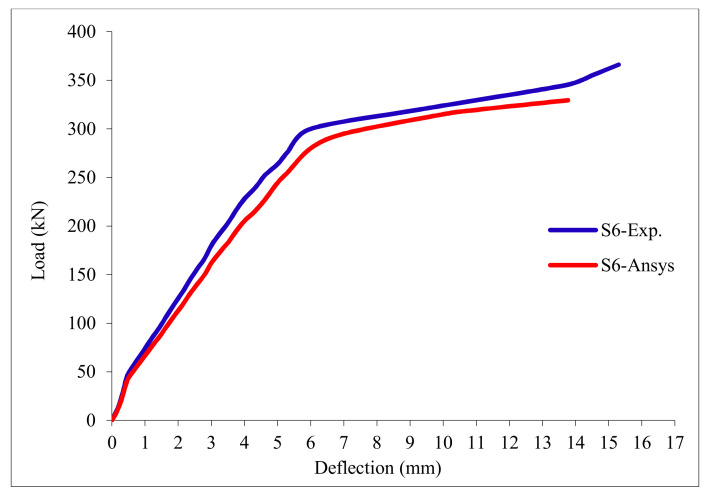
Load deflection curve for control slab S6.

Table 13 & Figure 25 shows the comparison between the ultimate experimental displacement and the numerical one. The load-displacement curves for experimental and modeled slabs, as shown in Figure 26, Figure 27, Figure 28, Figure 29, Figure 30 and Figure 31, showed good agreement with respect to control slab deflection. The impact of RSA with 15% on slab load-carrying capacity is clear from the result.

### 5.3. Cracking Patterns 

**Figure 32:**
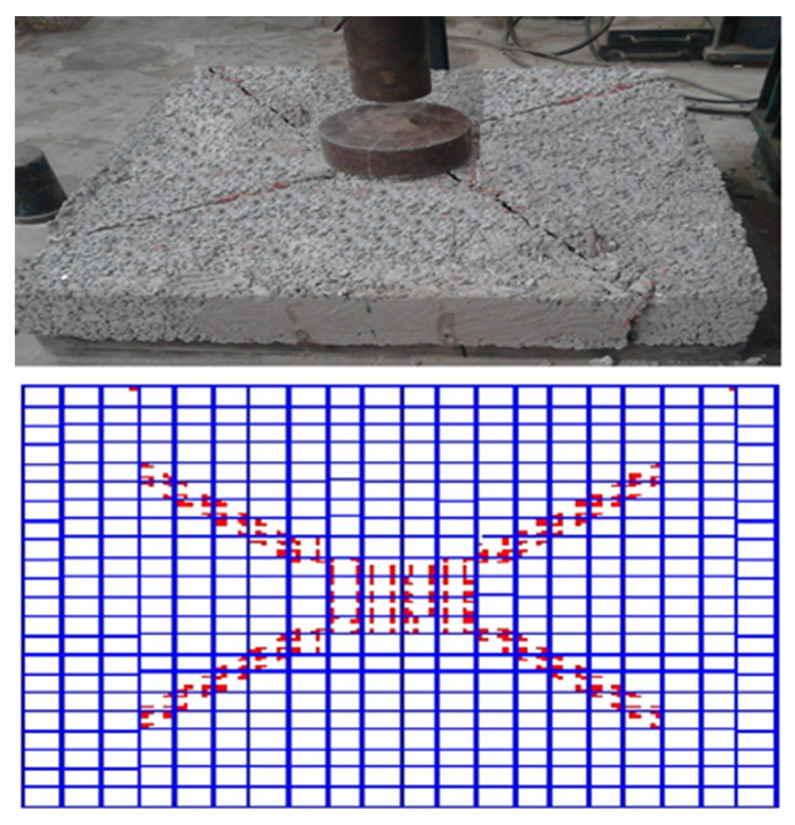
Crack spread for control specimen.

Figure 32 shows the typical cracking patterns of cracks for all reinforced pervious slabs obtained from experimental and nonlinear studies. The tension crack first appeared in slabs, and, with an increase in the load, the cracks widened and extended towards the compression zone. The slab failed by a diagonal crack under the loading plate.

## 6. Conclusions

Based on the studies performed on the pervious concrete prepared from normal and recycled materials, the following points were drawn as follows:

1. Rice and wheat straw ash gave accepted permeabilities and porosities with lower densities compared to the control mixes. This was attributed to the chemical compositions for rice and wheat straw ash.
2. Rice and wheat straw ash increased the compaction factor due to their spherical shapes and higher surface area compared with the control mixes.
3. Increasing the cement/aggregate ratio from 0.22 to 0.40 led to increases in the 28-day compressive strength, splitting tensile strength, and flexural strength.
4. Using sand increased the compressive, splitting tensile and flexural strengths, with a significant decrease in porosity and permeability. This was attributed to the higher density, which reduced the void content. Thus, we recommend the use of sand with a percentage less than 10% for accepted permeability.
5. The results showed that permeability and porosity increased slightly for mixes using rice and wheat straw ash. This could be attributed to increasing the interconnected voids. Optimum porosity was reached with 15% rice straw ash (Mix 7).
6. The concrete mixes with higher porosity showed high water permeability and low compressive splitting tensile and flexural strengths. The results proved that porosity and compressive strength were in a reverse relationship.
7. The void ratio should be controlled by employing polypropylene fibers, such as E300, to control the gap between the particles; consequently, the compressive strength will be increased.
8. All the studied reinforced pervious concrete slabs provided an increase in ultimate loads and displacements compared to the unreinforced control slab.
9. Pervious slabs reinforced by GFRP bars gave the highest ultimate load, followed by the slabs reinforced with geogrid, while the lowest ultimate load was for slabs with polypropylene fibers.
10. A nonlinear study was performed to verify the experimental study. The numerical results were in good agreement with the experimental ones.

Finally, the presented study showed that the green recycled pervious concrete provided good economic savings compared with the other types. In addition, this concrete could be used for sustainability purposes.

## Figures and Tables

**Table 1:** Concrete mix design for Phase I.

Item	Mix No.	Cement (kg/m^3^)	Coarse Aggregate (kg/m^3^)	Fine Aggregate (kg/m^3^)	Rice Straw Ash (kg/m^3^)	Wheat Straw Ash (kg/m^3^)	Water (kg/m^3^)	W/B	Superplasticizer (kg/m^3^)
Per m^3^ of concrete	M1	400	1750	---	---	---	95	0.238	2
	M2	400	1167	---	---	---	95	0.238	2
	M3	400	900	---	---	---	95	0.238	2
	M4	400	900	100	---	---	95	0.238	2
	M5	400	900	150	---	---	95	0.238	2
	M6	360	900	---	40	---	95	0.238	2
	M7	340	900	---	60	---	95	0.238	2
	M8	360	900	---	---	40	95	0.238	2
	M9	340	900	---	---	60	95	0.238	2

**Table 2:** Physical properties of the sand.

Property	Results	EES Acceptance Limits *
Specific gravity (kg/m^3^)	2.50	−
Unit weight (kg/m^3^)	1620	−
Materials finer than no. 200 sieve (0.074 mm) %	1.4	Less than 4%

**Table 3:** The physical and mechanical properties of the coarse aggregate.

Property	Value	EES Acceptance Limits *
Specific gravity (kg/m^3^)	2.60	−
Unit weight (kg/m^3^)	1600	---
Absorption Percentage	1.46%	Not more than 2.5%
Clay and other fine materials (%)	0.28%	Not more than 3% by weight
Abrasion value (loss angles) (%)	31.03%	Not more than 30%
Crushing value (%)	18.93%	Not more than 45%
Impact value (%)	----	Not more than 45%
Flakiness Index (%)	21.33%	Not more than 25%
Elongation Index (%)	4.33%	Not more than 25%

**Table 4:** The physical properties of the cement.

Property		OPC
Fineness (cm^2^/g)		3550
Setting time (min)	Initial	135
	Final	195
Soundness (mm)		1
Compressive strength (N/mm^2^)	2 days	26.2
	28 days	48.6

**Table 5:** Chemical analysis of the cement.

Component	SiO_2_	Al_2_O_3_	Fe_2_O_3_	CaO	Na_2_O	MgO	K_2_O	SO_2_	Loss on Ignition	Insoluble Residue
Percent	21	6.1	3	61.5	0.38	2.1	0.3	2.5	2.4	0.9

**Table 6:** The chemical composition of rice straw ash (RSA) and wheat straw ash (WSA).

Oxides	SiO_2_	Al_2_O_3_	Fe_2_O_3_	CaO	MgO	Na_2_O	K_2_O	SO_3_
RSA	91.33	0.07	0.07	0.45	0.28	0.01	2.64	0.05
WSA	88.32	0.06	0.06	0.67	0.44	0.07	1.89	0.14

**Table 7:** Physical and mechanical properties of polypropylene fibers.

Specific Gravity	0.91 gm/cm^3^
Thickness of the package	2 mm
Each fiber bandle	10
Tensile strength	370 N/mm^2^
Young’s modulus	3750 N/mm^2^
Acid and Salt Resistance	High
Alkali Resistance	Alkali Proof
Surface resistance	>10^3^ ohm
Volumetric resistance	>10^3^ ohm
Electrical Conductivity	Low
Melting Point	160 °C
Ignition Point	>320 °C

**Table 8:** Tensile strength and ultimate strains.

Diameter (mm)	Tensile Strength (MPa)	Strain (mm/mm)
10	740	0.0120

**Table 9:** Concrete mix design for Phase II.

Item	Cement (kg/m^3^)	Coarse Aggregate (kg/m^3^)	Rice Straw Ash (kg/m^3^)	Water (kg/m^3^)	W/B	Superplasticizer (kg/m^3^)
Per m^3^ of concrete	340	900	60	95	0.238	2

**Table 10:** Slab notation.

Series	Slab ID	Slab Description	Volume of Fraction (mm^3^)
Control	S1	Control	---
Ploy. fibers	S2	0.1% ploy. fibers	0.00270
	S3	0.2% ploy. fibers	0.00540
Geogrid	S4	CE121 type I	0.00753
	S5	CE121 type II	0.01510
GFRP bars	S6	5φ10/m’ bars both sides	---

**Table 11:** Experimental results.

Slab No	First Crack Load (kN)	Ultimate Load (kN)	Def. at First Crack Load (mm)	Def. at Ult. Load (mm)	Ductility Ratio %	Energy Absorption (kN.mm)
S1	45.10	155.30	3.45	12.10	3.48	932.58
S2	53.40	179.80	3.53	14.50	4.11	1303.55
S3	55.70	180.20	3.63	13.90	3.83	1252.39
S4	72.20	352.80	4.22	19.40	4.60	3422.16
S5	61.50	319.00	3.84	17.10	4.45	2727.45
S6	77.80	366.10	4.91	15.30	3.12	2800.67

**Table 12:** Numerical results.

Slab No	First Crack Load (kN)	Ultimate Load (kN)	Def. at First Crack Load (mm)	Def. at Ult. Load (mm)	Ductility Ratio	Energy Absorption (kN.mm)
S1	42.00	139.77	3.11	10.89	3.50	761.05
S2	49.00	161.82	3.42	13.05	3.82	1055.88
S3	51.00	162.18	3.59	12.51	3.48	1014.44
S4	63.00	317.52	4.18	17.46	4.18	2771.95
S5	59.00	287.10	3.81	15.39	4.04	2209.23
S6	75.00	329.49	4.87	13.77	2.83	2268.54

**Table 13:** Experimental and numerical results.

Slab No	First Crack Load (kN)	First Crack Load (kN)	Ultimate Load (kN)	Ultimate Load (kN)	Def. at Ult. Load (mm)	Def. at Ult. Load(mm)
	NLA.	EXP.	NLA.	EXP.	NLA.	EXP.
S1	42.00	45.10	139.77	155.30	10.89	12.01
S2	49.00	53.40	161.82	179.80	13.05	14.50
S3	51.00	55.70	162.18	180.20	12.51	13.90
S4	63.00	72.20	317.52	352.80	17.46	19.40
S5	59.00	61.50	287.10	319.00	15.39	17.10
S6	75.00	77.80	329.49	366.10	13.77	15.30

## Data Availability

Not applicable.

## Abbreviations

OPC	Ordinary Portland Cement
ACI	American Concrete Institute
ASTM	American Society for Testing and Materials
WSA	Wheat Straw Ash
RSA	Rice Straw Ash
W/C	Water/Cement
W/B	Water/Binder
CSH	Calcium silicate hydrate
CAH	Calcium Aluminate Hydrate
CEM	Cement
BASF	Badische Anilin- und Soda-Fabrik
ESS	Egyptian Standard Specifications for the design and construction of reinforced concrete structures (2018)
Exp.	Experimental
NLA	Nonlinear analysis

## References

[1] ACI 522R-10 (2010). Report on Pervious Concrete.

[2] Bentz D.P. (2008). Virtual pervious concrete: Microstructure, percolation, and permeability. ACI Mater. J..

[3] Schaefer V.R., Wang K. (2006). Mix Design Development for Pervious Concrete in Cold Weather Climates.

[4] Li L., Aubertin M. (2003). A general relationship between porosity and uniaxial strength of engineering materials. Can. J. Civ. Eng..

[5] Lian C., Zhuge Y., Beecham S. (2011). The relationship between porosity and strength for pervious concrete. Constr. Build. Mater..

[6] Wang K., Schaefer V., Kevern J., Suleiman M. (2006). Development of mix proportion for functional and durable pervious concrete. Development of Mix Proportion for Functional and Durable Pervious Concrete.

[7] Maguesvari M.U., Narasimha V. (2013). Studies on characterization of pervious concrete for pavement applications. Procedia Soc. Behav. Sci..

[8] Lian C., Zhuge Y. (2010). Optimum mix design of enhanced permeable concrete—An experimental investigation. Constr. Build. Mater..

[9] Bonicelli A., Giustozzi F., Crispino M. (2015). Experimental study on the effects of fine sand addition on differentially compacted pervious concrete. Constr. Build. Mater..

[10] Fu T.C., Yeih W., Chang J.J., Huang R. (2014). The influence of aggregate size and binder material on the properties of pervious concrete. Adv. Mater. Sci. Eng..

[11] Yang J., Jiang G. (2003). Experimental study on properties of pervious concrete pavement materials. Cem. Concr. Res..

[12] Crouch L., Pitt J., Hewitt R. (2007). Aggregate effects on pervious portland cement concrete static modulus of elasticity. J. Mater. Civ. Eng..

[13] Bhutta M.A.R., Tsuruta K., Mirza J. (2012). Evaluation of high-performance pervious concrete properties. Constr. Build. Mater..

[14] Joshaghani A., Ramezanianpour A.A., Ataei O., Golroo A. (2015). Optimizing pervious concrete pavement mixture design by using the Taguchi method. Constr. Build. Mater..

[15] Mohammed B.S., Liew M.S., Alaloul W.S., Khed V.C., Hoong C.Y., Adamu M. (2018). Properties of nano-silica modified pervious concrete. Case Stud. Constr. Mater..

[16] Gao J., Qian C., Wang B., Morino K. (2002). Experimental study on properties of polymer-modified cement mortars with silica fume. Cem. Concr. Res..

[17] Aggarwal L., Thapliyal P., Karade S. (2007). Properties of polymer-modified mortars using epoxy and acrylic emulsions. Constr. Build. Mater..

[18] Ariffin N.F., Hussin M.W., Sam A.R.M., Bhutta M.A.R., Khalid N.H.A., Mirza J. (2015). Strength properties and molecular composition of epoxy-modified mortars. Constr. Build. Mater..

[19] Giustozzi F. (2016). Polymer-modified pervious concrete for durable and sustainable transportation infrastructures. Constr. Build. Mater..

[20] Huang B., Wu H., Shu X., Burdette E.G. (2010). Laboratory evaluation of permeability and strength of polymer-modified pervious concrete. Constr. Build. Mater..

[21] Shen W., Shan L., Zhang T., Ma H., Cai Z., Shi H. (2013). Investigation on polymer–rubber aggregate modified pervious concrete. Constr. Build. Mater..

[22] Bhutta M.A.R., Hasanah N., Farhayu N., Hussin M.W., Tahir M.b.M., Mirza J. (2013). Properties of pervious concrete from waste crushed concrete (recycled aggregate). Constr. Build. Mater..

[23] Chen Y., Wang K., Wang X., Zhou W. (2013). Strength, fracture and fatigue of pervious concrete. Constr. Build. Mater..

[24] Niu Q., Feng N., Yang J., Zheng X. (2002). Effect of superfine slag powder on cement properties. Cem. Concr. Res..

[25] Mahmud H.B., Chia B.S., Hamid N.B.A.A. Rice husk ash—An alternative material in producing high strength concrete. Proceedings of the International Conference on Engineering Materials.

[26] Zhang M.H., Malhotra V.M. (1996). High-performance concrete incorporating rice husk ash as a supplementary cementing material. ACI Mater. J..

[27] Senff L., Labrincha J.A., Ferreira V.M., Hotza D., Repette W.L. (2003). Effect of nano-silica on rheology and fresh properties of cement pastes and mortars. Constr. Build. Mater..

[28] El-Sayed T.A., Erfan A.M., Abd El-Naby R.M. (2017). Influence of Rice, Wheat Straw Ash & Rice Husk Ash on The properties of Concrete Mixes. Jokull.

[29] El-Sayed T.A., Erfan A.M., Abd El-Naby R.M. (2019). Recycled rice & wheat straw ash as cement replacement materials. J. Eng. Res. Rep..

[30] El-Sayed T.A., Erfan A.M., El-Naby R.M.A. (2019). Flexural Behavior of RC Beams by Using Agricultural Waste as a Cement Reinforcement Materials. J. Eng. Res. Rep..

[31] El-Sayed T.A., Shaheen Y.B. (2020). Flexural performance of recycled wheat straw ash-based geopolymer RC beams and containing recycled steel fiber. Structures.

[32] Chen Y., Wang K.J., Zhou W.F. (2013). Evaluation of surface textures and skid resistance of pervious concrete pavement. J. Cent. South Univ..

[33] Shen W., Shi H., Ma H., Zhang W., Lian C., Ye J. (2013). The Morphology of CSH in Low Water/Cement Ratio Paste from Nano to Micro Scale. Curr. Nanosci..

[34] Lee M., Huang Y., Chang T., Pao C. (2011). Experimental study of pervious concrete pavement. Emerging Technologies for Material, Design, Rehabilitation, and Inspection of Roadway Pavements.

[35] Rehder B., Banh K., Neithalath N. (2014). Fracture behavior of pervious concretes: The effects of pore structure and fibers. Eng. Fract. Mech..

[36] Amde A.M., Rogge S. (2013). Development of High Quality Pervious Concrete Specifications for Maryland Conditions.

[37] Zhong R., Wille K. (2016). Compression response of normal and high strength pervious concrete. Constr. Build. Mater..

[38] Cyr M., Lawrence P., Ringot E. (2006). Efficiency of mineral admixtures in mortars: Quantification of the physical and chemical effects of fine admixtures in relation with compressive strength. Cem. Concr. Res..

[39] Binici H., Aksogan O., Kaplan H. (2005). A study on cement mortars incorporating plain Portland cement (PPC), ground granulated blast-furnace slag (GGBFS) and basaltic pumice. Indian J. Eng. Mater. Sci..

[40] Christy C.F., Tensing D. (2010). Effect of Class-F fly ash as partial replacement with cement and fine aggregate in mortar. Indian J. Eng. Mater. Sci..

[41] Bonavetti V., Donza H., Rahhal V., Irassar E. (2000). Influence of initial curing on the properties of concrete containing limestone blended cement. Cem. Concr. Res..

[42] Pera J., Husson S., Guilhot B. (1999). Influence of finely ground limestone on cement hydration. Cem. Concr. Compos..

[43] Hameed M.S., Sekar A.S.S., Balamurugan L., Saraswathy V. (2012). Self-compacting concrete using marble sludge powder and crushed rock dust. KSCE J. Civ. Eng..

[44] Ergün A. (2011). Effects of the usage of diatomite and waste marble powder as partial replacement of cement on the mechanical properties of concrete. Constr. Build. Mater..

[45] Isaia G.C., GASTALDInI A.L.G., Moraes R. (2003). Physical and pozzolanic action of mineral additions on the mechanical strength of high-performance concrete. Cem. Concr. Compos..

[46] Phoo-ngernkhama T., Chindaprasirt P., Sata V., Sinsiri T. (2013). High calcium fly ash geopolymer containing diatomite as additive. Indian J. Eng. Mater. Sci..

[47] ESS 203-2018 (2018). Egyptian Standard Specifications for the Design and Construction of Reinforced Concrete Structures.

[48] ASTM C 127-88 (1993). Standard Test Method for Specific Gravity and Absorption of Coarse Aggregate.

[49] ASTM C136-84a (1998). Standard Test Method for Sieve Analysis of Fine and Coarse Aggregate.

[50] ASTM C150/C150M (2018). Standard Specification for Portland Cement.

[51] ASTM C618 (2017). Standard Specification for Coal Fly Ash and Raw or Calcined Natural Pozzolan for Use in Concrete.

[52] Bong S.H., Nematollahi B., Nazari A., Xia M., Sanjayan J. (2019). Efficiency of different superplasticizers and retarders on properties of ‘one-Part’ Fly ash-slag blended geopolymers with different activators. Materials.

[53] ASTM C143/C 143 M-12-05a (2005). Standard Test Method for Slump of Hydraulic Cement Concrete.

[54] ACI Committee 211.3R-02 (1974). Standard Practice for Selecting Proportions for No-Slump Concrete.

[55] ASTM C138/ C138 M-13-10-01a (2017). Standard Test Method for Density (Unit Weight), Yield and Air Content (Gravimetric) of Concrete.

[56] ASTM C39/ C39M-14 (2014). Standard Test Method for Compressive Strength of Cylindrical Concrete Specimens.

[57] ASTM C 496/C 496M (2004). Standard Test Method for Splitting Tensile Strength of Cylindrical Concrete Specimens.

[58] ASTM C78 /C78-M-10-02 (2002). Standard Test Method for Flexural Strength of Concrete Using Simple Beam with Third-Point Loading.

[59] ASTM PS 129 (2001). Standard Provisional Test Method for Measurement of Permeability of Bituminous Paving Mixtures Using a Flexible Wall Permeameter.

[60] ASTM D7063/D7063M (2011). Standard Test Method for Effective Porosity and Effective Air Voids of Compacted Bituminous Paving Mixture Samples.

[61] Mazumdar A.R., Boonsriarporn K., Kongseng S., Minsar M.A.M. (2018). Pervious Concrete and the Effect of Supplementary Cementitious and Fine Aggregate on its Performance. Adv. Concr. Technol..

[62] Desmaliana E., Herbudiman B., Saputra W.Y. Mechanical properties of pervious concrete with variations of coarse aggregate gradation. Proceedings of the MATEC Web of Conferences.

[63] Jankovský O., Pavlikova M., Sedmidubský D., Bouša D., Lojka M., Pokorný J., Zaleska M., Pavlík Z. (2017). Study on pozzolana activity of wheat straw ash as potential admixture for blended cements. Ceram. Silikáty.

[64] Shagea A., Smit K. (2017). Effects of Cement Kiln Dust on the Properties of Pervious Concrete. IJSRD.

[65] Narayana D., Shariff S.A., Ahmed N., Ahmed U., Rehman S. (2019). Experimental Investigation on the Properties of Pervious Concrete Over Fiber-Reinforced Pervious Concrete. Sustainable Construction and Building Materials.

[66] Kia A., Wong H.S., Cheeseman C.R. (2017). Clogging in permeable concrete: A review. J. Environ. Manag..

[67] Erfan A.M., Abd Elnaby R.M., Badr A.A., El-sayed T.A. (2021). Flexural behavior of HSC one way slabs reinforced with basalt FRP bars. Case Stud. Constr. Mater..

[68] Roesler J.R., Altoubat S.A., Lange D.A., Rieder K.A., Ulreich G.R. (2006). Effect of synthetic fibers on structural behavior of concrete slabs-on-ground. ACI Mater. J..

[69] Mahroug M.E.M., Ashour A.F., Lam D. (2014). Tests of continuous concrete slabs reinforced with carbon fibre reinforced polymer bars. Compos. Part B.

[70] ANSYS (2005). Engineering Analysis System User’s Manual and Theoretical Manual.

